# Early intervention of acute liver injury related to venlafaxine: A case report

**DOI:** 10.1097/MD.0000000000028140

**Published:** 2021-12-10

**Authors:** Lin Fang, Shushan Wang, Leiming Cao, Kun Yao

**Affiliations:** a Department of Clinical Psychology, The Affiliated Wuxi Mental Health Center with Nanjing Medical University, Wuxi, Jiangsu 214151, China; b Department of Pharmacy, The Affiliated Wuxi Mental Health Center with Nanjing Medical University, Wuxi, Jiangsu 214151, China.

**Keywords:** acute liver injury, drug-induced liver injure, causality assessment, venlafaxine

## Abstract

**Rationale:**

Drug-induced liver injury (DILI) is the leading cause of acute liver injury (ALI), market withdrawal of a drug, and rejection of applications for marketing licenses. The incidence of DILI is very low, with a value between 1 and 19 per 100,000 patient years. All antidepressants may induce DILI even at low therapeutic doses. In this report, we present a case of ALI after venlafaxine administration.

**Patient concerns:**

A 27-year-old Chinese Han woman was admitted for depression. Several serum liver function indices in this patient were abnormal after antidepressant treatment. The Roussel Uclaf Causality Assessment Method (RUCAM) causality assessment score was 8, and the *R* value was 31.18.

**Diagnoses:**

The patient was diagnosed with hepatocellular ALI, which was derived from venlafaxine-related adverse events.

**Interventions:**

First, all medications were stopped to block the progression of DILI. Then, a hepatoprotective strategy and proper psychological treatment were performed to recover the impaired hepatic function.

**Outcomes:**

Liver function was fully recovered as indicated by liver function indices and ultrasound imaging.

**Lessons:**

The possibility of DILI should not be overlooked during the long-term use of antipsychotic drugs. In response, regular liver function monitoring should be performed in a timely manner to avoid missing diagnoses and delayed treatment. Furthermore, the necessary medical treatment needs to be conducted after the occurrence of ALI.

## 1 Introduction

As a serotonin and norepinephrine reuptake inhibitor, venlafaxine (VEN) has been proven to be well tolerated with a rate of adverse drug reactions (ADRs) of less than 1/1000.^[1]^ The most common ADRs^[2,3]^ are mild, including nausea, vomiting, dizziness, diarrhea, dry mouth, decreased appetite, constipation, somnolence, and so on, all of which might be caused by serotonin toxicity or vulnerability to drug-drug interactions.^[4]^ Notably, VEN-induced acute liver injury (ALI) has been less reported since its approval in 1993.^[5–7]^ It has been confirmed that VEN can result in transient asymptomatic elevations in serum aminotransferase levels and has been linked to rare instances of clinically apparent ALI.^[8]^ However, the mechanism by which VEN causes ALI is unknown. In this case report, we describe 1 patient with depression who was diagnosed with ALI after the administration of VEN.

## 2 Case presentation

A 27-year-old Chinese Han woman was diagnosed with depression. She had no chronic or metabolic liver disease, blood transfusion, or history of alcohol consumption. The patient was admitted to our Department of Clinical Psychology on March 9, 2021, and low-dose VEN 50 mg (oral, qd) was started to give to her. Meanwhile, liver function was normal according to the test results (Table 1). The dosage of VEN was gradually increased to 225 mg/d on March 19 because of severe depression. The patient showed good tolerance without any symptoms. Additionally, she also received lorazepam 1 mg (oral, qn) and zolpidem tartrate 10 mg (oral, qn) for treating anxiety symptoms and dyssomnia during this period. After administering these treatments, depressive symptoms were well controlled.

**Table 1:** The results of liver function tests and the reference values.

Aminotransferase	March 10	April 14	April 19	April 29	Reference value
AST	13 IU/L	767 IU/L	24 IU/L	17 IU/L	5–50 IU/L
ALT	12 IU/L	1777 IU/L	269 IU/L	37 IU/L	7–40 IU/L
ALP	39 IU/L	171 IU/L	85.5 IU/L	57.0 IU/L	40–150 IU/L
γ-GT	7 IU/L	147 IU/L	90 IU/L	48 IU/L	7–50 IU/L
5′-nucleotidease	2.9 U/L	37 U/L	12 U/L	5.3 U/L	2–11.4 U/L

**Figure 1:**
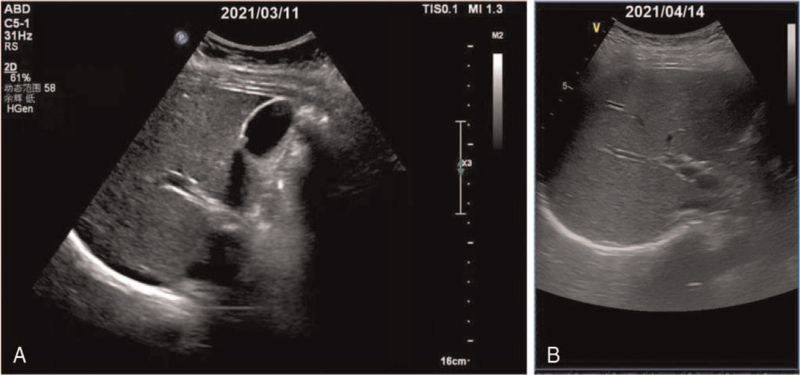
The ultrasound of the liver on March 11, 2021 (A) and April 14, 2021 (B). There was no dilatation of the intrahepatic or extrahepatic bile ducts and hepatomegaly or splenomegaly appeared in 2 images.

Before leaving the hospital, the liver function as well as her blood routine were reviewed on April 14, 2021. The results of routine blood tests were normal, while the symptoms of ALI (Table 1) were abnormal elevation of serum aspartate aminotransferase to 767 IU/L, alanine aminotransferase to 1777 IU/L, alkaline phosphatase to 171 IU/L, γ-glutamyltransferase to 147 IU/L, and 5’-nucleotidease to 37 U/L. Nevertheless, neither dilatation of the intrahepatic or extrahepatic bile ducts nor hepatomegaly or splenomegaly were detected by ultrasound of the liver (Fig. 1). These results indicate that ALI was still in its early stages. The results of Roussel Uclaf Causality Assessment Method (RUCAM) assessment (score: 8) illustrated that this ALI (hepatocellular, *R* = 31.18) was derived from VEN-related adverse events.

Based on these considerations, stopping all medications was adopted to block the progression of ALI. g of glutathione 1.08 g of magnesium isoglycyrrhizinate 150 mg (intravenous, qd) and silibinin meglumine 100 mg (oral, tid) were administered to the patient. Several indices of liver function were recovered based on the results from April 19, 2021 (Table 1). Therefore, the current hepatoprotective strategy, as well as proper psychological treatment, were held for this patient. The liver function fully recovered on April 29, 2021 (Table 1), and she was discharged from the hospital the next day.

## 3 Discussion

ALI is a rare manifestation of VEN-induced ADRs. Despite the unknown mechanism, we attempted to reveal the pathogeny of VEN-related ALI in this case. It has been reported that the dosage of VEN-related liver function abnormalities is in the range of 25 to 300 mg/d with a median dose of 75 mg/d, and the upper limit of normal for concentration of VEN in the patients’ plasma is 400 ng/mL.^[9]^ It is critical to note that this upper value may be exceeded in patients who receive only the conventional dosage. This individual difference increases the risk of dose-related adverse events. In view of these facts, we firstly performed the plasma drug concentration measurements and the result was only 93.9 ng/mL, which was even slightly lower than the recommended therapeutic concentration of Arbeitsgemeinschaft für Neuropsychopharmakologie und Pharmakopsychiatrie (100–400 ng/mL). Second, we attempted to determine the metabolic features of VEN in this patient. Cytochrome P450 (CYP) 2D6 in the liver is the predominant metabolic enzyme responsible for converting 90 percent of VEN to O-desmethylvenlafaxine (desvenlafaxine).^[10]^ Additionally, CYP2C19 mediates the remaining 10% of this demethylation. Indeed, extensive CYP2D6 and CYP2C19 metabolizers always increase the clearance of VEN and maintain a low plasma concentration. To our surprise, results of genetic polymorphism testing showed that CYP2D6 was a normal metabolizer with rs1065852 CT, rs16947 CT, and rs5030865 GG, while CYP2C19 was an extensive metabolizer with rs4244285 GG, rs4986893 GG, and rs12248560 CC, which is in accordance with the low exposure of VEN. Third, the vulnerability of VEN to drug-drug interactions can also cause ADRs. In this case, the fast metabolic characteristics of VEN lead to a high abundance of cytochrome isoenzymes (mainly CYP2D6 and CYP2C19) as well as rapidly accumulated O-desmethylvenlafaxine, which are thought to be involved in VEN-associated liver injury.

As a reversible profile of hepatic damage, accurate and timely liver function monitoring and the necessary measures taken after abnormalities in the liver are crucial to recover its function. Corticosteroids, such as methylprednisolone, are used as hepatic protectants against VEN-induced liver injury.^[11]^ As another liver cell protective agent, magnesium isoglycyrate always plays a hepatoprotective effect by protecting the liver cell membrane, improving liver function, and its anti-inflammatory ability. It can inhibit the increase in serum transaminase, reduce the degeneration, necrosis, and inflammatory cell infiltration of liver cells, and hence repair the liver tissue and recover its activity.^[12–15]^ In addition, VEN was shown to induce ROS formation to enhance the membrane permeability of mitochondria and lysosomes, triggering apoptosis or necrosis.^[16]^ Thus, ROS scavenger agents have been identified as promising therapeutic strategies against drug/xenobitic-induced liver injuries.^[17]^

## 4 Conclusion

During the administration of VEN or other antidepressants, liver function monitoring is crucial, and immediate drug withdrawal as well as necessary medical treatment should be taken in cases of ALI to obtain satisfactory post-treatment results. Furthermore, more samples and further explorations are still needed to better understand the etiology, early diagnosis, and treatment of VEN-induced ALI. We hope that our report will provide an effective reference for future research on this DILI.

## Author contributions

**Conceptualization:** Lin Fang, Kun Yao.

**Funding acquisition:** Kun Yao.

**Writing – original draft:** Lin Fang, Shushan Wang.

**Writing – review & editing:** Leiming Cao, Kun Yao.
